# Consumers’ Needs for Laboratory Results Portals: Questionnaire Study

**DOI:** 10.2196/42843

**Published:** 2023-06-12

**Authors:** Helen Monkman, Janessa Griffith, Leah MacDonald, Blake Lesselroth

**Affiliations:** 1 School of Health Information Science University of Victoria Victoria, BC Canada; 2 Health Information Management Douglas College Coquitlam, BC Canada; 3 Work Wellness Institute Vancouver, BC Canada; 4 Department of Medical Informatics School of Community Medicine University of Oklahoma Tulsa Tulsa, OK United States

**Keywords:** consumer health information, user-centered design, clinical laboratory information systems, laboratory test result, patient portal, laboratory result, facilitator, barrier, information system, questionnaire, usability

## Abstract

**Background:**

Over the last decade, there has been an increase in the number of health care consumers (ie, patients, citizens, and laypeople) with access to their laboratory results through portals. However, many portals are not designed with the consumer in mind, which can limit communication effectiveness and consumer empowerment.

**Objective:**

We aimed to study design facilitators and barriers affecting consumer use of a laboratory results portal. We sought to identify modifiable design attributes to inform future interface specifications and improve patient safety.

**Methods:**

A web-based questionnaire with open- and closed-ended items was distributed to consumers in British Columbia, Canada. Open-ended items with affinity diagramming and closed-ended questions with descriptive statistics were analyzed.

**Results:**

Participants (N=30) preferred reviewing their laboratory results through portals rather than waiting to see their provider. However, respondents were critical of the interface design (ie, interface usability, information completeness, and display clarity). Scores suggest there are display issues impacting communication that require urgent attention.

**Conclusions:**

There are modifiable usability, content, and display issues associated with laboratory results portals that, if addressed, could arguably improve communication effectiveness, patient empowerment, and health care safety.

## Introduction

Ambulatory care practitioners (ie, primary care providers and medical specialists) frequently order laboratory tests for patients as part of a diagnostic evaluation or to monitor the progression of chronic illness [1]. In the past, practitioners received paper reports with test results, whereas now, it is more common to review results in the electronic health record (EHR). Commercial EHR developers assume the target users have sufficient domain expertise to access and use this information with little additional context or instruction.

However, health care consumers (ie, patients, citizens, and laypeople) are increasingly accessing their own laboratory results (eg, COVID-19, Papanicolaou smear, and blood work results) through independent laboratory portals or patient-facing portals tethered to an organizational EHR. Research shows that people want access to their laboratory results [2-4] to track their health status and guide decision-making [4,5]. Those with chronic illnesses can use this information to monitor [6] and more effectively self-manage their own medical conditions [4,7]. Having direct access to laboratory results through portals often means getting results sooner [3,4] and without the inconvenience of scheduling a follow-up appointment or traveling to see a health care practitioner [6]. Furthermore, consumers with results in hand are empowered to engage more effectively in a discussion with clinicians during appointments [2,5] and ensure results are not overlooked [4,6].

It is imprudent, however, to equate access with value; just because consumers can see information does not mean they can understand or use it. Several studies of consumer portals have found that while Canadians appreciate being able to access information on the internet, they struggle to understand and use their results [3,8]. The introduction of new technologies can alter traditional workflows. Circumventing in-person appointments—and the explanations or education practitioners provide during these encounters—may limit communication effectiveness or produce unintended consequences. Laboratory results and technical reports can be complicated and difficult to interpret without medical expertise and additional context.

Researchers have identified several shortcomings in the display of laboratory results portals that reduce their usefulness as patient communication, education, or self-management tools. For example, Leckart [9] noted that laboratory results are typically long text-based reports with many unfamiliar acronyms. The reports also separate patient values from associated reference ranges. By contrast, using graphs to depict values with reference ranges improves consumers’ ability to interpret results [10]. While the need to redesign laboratory reports was well documented over a decade ago [9], little progress was made to include emphasis cues, contextual information, or hypertext links to related resources. Consumers want their test results combined with actionable information [11,12]. Unfortunately, laboratory results portals rarely include context-sensitive interpretation [11] or recommendations to improve values [9]. These challenges are compounded for Canadians with low health literacy [13]. In light of these issues, it is unsurprising that nearly half (46%) of consumers turn to the internet to find answers to their questions about laboratory results [11].

Further complicating matters, consumers may need to use multiple different portals to review all their information. Health care provider organizations within a community of practice may use a tapestry of different EHR vendors, products, and features. Consequently, not only are the data fragmented between different systems, but users may have different user experiences (eg, button locations and information displays) depending on where the laboratory tests were ordered or processed. In Canada, some consumers have had access to some of their laboratory results portals for over a decade. For example, in British Columbia, independent laboratory results have been on the internet since 2010 [3]. Canadians may now also access laboratory results performed during hospitalizations using patient portals tethered to an EHR. However, the information remains siloed; laboratory results are only accessible through the portal linked to where the laboratory tests were done (ie, ambulatory independent laboratory vs hospital). Therefore, the information is fragmented for users.

The purpose of this study was to identify interface usability issues and associated modifiable attributes of laboratory results portals (including more comprehensive portals tethered to EHRs). Our goal was to uncover design strategies that might inform future portal specifications and improve the communication of laboratory results. We provided a questionnaire to a sample of Canadians asking about their use of laboratory results portals, their perceptions of existing portals, and their perspectives on the design of information displays. Our inquiry was focused on general features related to laboratory results portal systems rather than a specific vendor, product, or health provider organization.

## Methods

### Study Design

We recruited people by posting an invitation on a web-based platform for health research volunteers in British Columbia, Canada. Participants accessed the questionnaire using a hypertext link; administration was unmoderated. To be eligible, participants (1) needed to have experience using at least one laboratory results portal and (2) be at least 19 years old. Health care professionals or trainees were excluded. Participants were offered CAD $5 (approximately US $3.70) as an honorarium to participate. The questionnaire was available from November 2020 to February 2021 (Multimedia Appendix 1).

In addition to gathering demographic information (eg, age, country of birth, and primary language spoken at home), we asked consumers about their experiences using laboratory results portals (eg, how long they had been using laboratory results portals) and their perceptions of the user experience (eg, usability, understandability, and information needs). Closed-ended Likert-type questions were used (1 star to 5 stars) to measure perceptions of user-friendliness (ie, usability), the available information (ie, content), and display formatting. We also included one question asking, “Would you suggest someone else use a lab results portal?” or a modified Net Promoter Score (NPS) [14] using a 5-star scale. Open-ended (ie, free-form response) questions were included to encourage participants to provide additional context or explanation. Participants were asked to provide general comments about laboratory results portals and specific suggestions for improving (1) the use of laboratory results portals, (2) the information (ie, numbers and words) contained in laboratory results portals, and (3) the laboratory results portal displays (eg, color and format).

### Ethics Approval

The University of Victoria’s Human Research Ethics Board approved this study (20-0712).

### Quantitative and Qualitative Analysis

Descriptive statistics were used to examine responses to the closed-ended questions. The modified NPS was calculated by first categorizing responses and calculating percentages for each category (ie, 1-3=detractors, 4=passive, and 5=promoters). We then subtracted the percentage of detractors from the percentage of promoters [14].

To analyze the qualitative data, we used affinity diagramming (ie, affinity mapping) to identify commonalities between responses [15,16]. Affinity diagramming is a common qualitative research method used to organize findings (eg, comments and observations) into groups that share semantic meaning or concepts [16]. The researchers (HM and LM) met over the web using Zoom videoconferencing software (Zoom Video Communications) to screen share and Microsoft PowerPoint to visualize and categorize each participant’s response. The responses to each open-ended question were analyzed separately. In cases where a participant’s response contained more than one concept, we separated the response into as many independent concepts as necessary. Each concept was also color-coded to indicate whether the content was positive, negative, or a suggestion for improvement. The groups of comments that emerged, reflecting the thematic similarities, were named. Some of these categories were hierarchical with subcategories. Finally, the content in each category was synthesized into a summary description.

After affinity diagramming, we compared our inductively coded categories to themes in the literature [17]. We replicated the coding from the affinity diagramming using MaxQDA (Verbi) qualitative analysis software to count the frequency with which each category was mentioned by participants.

## Results

### Descriptive Statistics

In total, 30 people completed the questionnaire (ie, N=30). Most participants were between 45 years or older (n=17, 57%), women (n=26, 87%) and born in Canada (n=23, 77%); spoke English at home (n=29, 97%); and had at minimum some post graduate training (eg, certificate, Bachelor’s degree) (n=16, 53%) (Table 1). Nearly three-quarters (n=22, 73%) of the participants had at least 1 chronic condition. The most common conditions reported included cardiovascular disease (n=6, 20%), mental illness (n=7, 23%), and musculoskeletal disorders (n=7, 23%). Most participants (n=25, 83%) took one or more prescription medications in the past 2 days.

There was variability in the amount of laboratory tests respondents had done and their use patterns of laboratory results portal use (see Table 2).

**Table 1:** Demographic characteristics of the sample.

Demographic characteristic		Value, n (%)
**Age (years)**		
	19-24	1 (3)
	25-34	8 (27)
	35-44	4 (13)
	45-54	7 (23)
	55-64	6 (20)
	65-74	4 (13)
**Gender**		
	Women	26 (87)
	Men	3 (10)
	Prefer not to disclose	1 (3)
**Country of birth**		
	Canada	23 (77)
	Other	7 (23)
**Primary language spoken at home**		
	English	29 (97)
	Other	1 (3)
**Highest level of education**		
	Secondary (high) school diploma or equivalency certificate	2 (7)
	Trades certificate or diploma other than Certificate of Apprenticeship or Certificate of Qualification	3 (10)
	College, College of General and Professional Teaching, or other nonuniversity certificate or diploma	4 (13)
	University certificate or diploma below bachelor level	5 (17)
	Bachelor’s degree	8 (27)
	University certificate or diploma above bachelor level	2 (7)
	Master’s degree	5 (17)
	Doctoral degree	1 (3)
**Chronic illnesses**		
	Yes	22 (73)
	No	8 (27)
**Type of chronic illnesses**		
	Musculoskeletal disorder	9 (30)
	Neurological condition	8 (27)
	Mental illness	8 (27)
	Cardiovascular disease	6 (20)
	Chronic respiratory disease	5 (17)
	Cancer	3 (10)
	Chronic pain	3 (10)
	Diabetes	2 (7)
	Other	8 (27)
**Number of prescription medications taken in the past 2 days**		
	None	5 (17)
	1	5 (17)
	2	3 (10)
	3	5 (17)
	4	3 (10)
	5+	9 (30)

**Table 2:** Descriptive statistics for participant use patterns.

Descriptive characteristic		Value, n (%)
**Last laboratory test done**		
	In the past month	17 (57)
	In the past 6 months	7 (23)
	In the past year	5 (17)
	In the past 5 years	1 (3)
**Frequency of getting laboratory tests done**		
	A few times a month	2 (7)
	A few times a year	21 (70)
	Once a year	5 (17)
	Less than once a year	2 (7)
**Laboratory results portals used**		
	Myehealth.ca (since renamed MyCareCompass)	28 (93)
	Other	8 (27)
**Started using laboratory results portals**		
	<1 year	1 (3)
	2-3 years	13 (43)
	4-5 years	6 (20)
	5+ years	10 (33)
**Frequency of using laboratory results portals**		
	A few times a month	5 (17)
	A few times a year	23 (77)
	Once a year	1 (3)
	Less than once a year	1 (3)

Most respondents first began using laboratory results portals (n=20, 67%) within the last 5 years and reviewed them several times a year (n=23, 77%). Nearly all respondents (n=29, 97%) reported using an independent laboratory portal available in the province (ie, MyeHealth.ca, which was recently renamed MyCareCompass), with 4 (13%) participants also using portals tethered to hospital EHRs. One (3%) participant reported only using a tethered hospital EHR laboratory results portal but not the independent portal.

### Overall Ratings of Laboratory Results Portals

Generally, participants rated laboratory results portals favorably but indicated opportunities for design improvements (Table 3). Most participants were very likely to recommend laboratory results portals to others (modified NPS=50; 19/30, 63.3% promoters – 4/30, 13.3% detractors). Participants scored usability the highest, followed by information, and then display. We review each dimension in the next section.

**Table 3:** Overall ratings of laboratory results portals.

Question topic	Question	Value, mean (SD)
Modified Net Promoter Score	Would you suggest someone else use laboratory results portals?	4.5 (0.73)
Usability	Overall, how user-friendly are your laboratory results portals?	3.7 (1.09)
Information	Overall, how would you rate the information (numbers and words) from laboratory results portals?	3.5 (0.73)
Display	Overall, how would you rate the display (layout, font size, color, etc) of laboratory results portals?	3.3 (0.60)

### Usability and Features of Laboratory Results Portals

#### Overview

**Figure 1:**
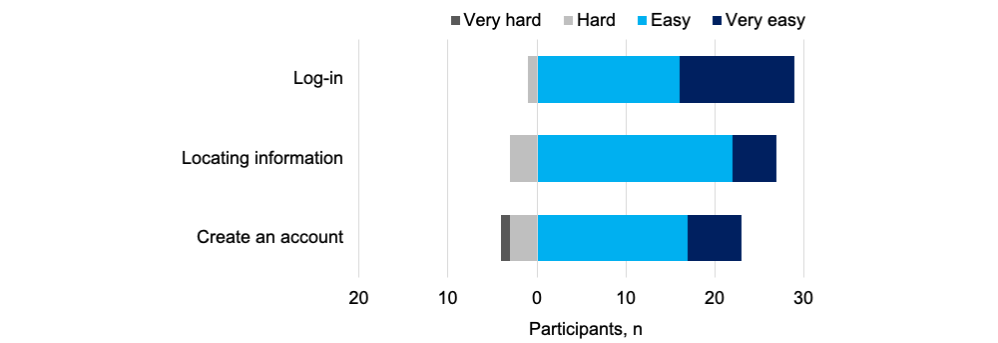
Perceived usability of laboratory results portal tasks.

Consistent with the overall usability rating (mean 3.7, SD 1.07; see Table 3), most participants indicated that creating an account (n=23, 77%), logging in (n=29, 97%), and finding information (n=28, 93%) was easy or very easy (Figure 1). Only 1 (3%) participant had difficulty logging into the portal, and 3 (10%) had trouble locating information. Participants had the most difficulty creating an account—4 (13%) said this was hard or very hard.

#### Features of Laboratory Results Portals

**Figure 2:**
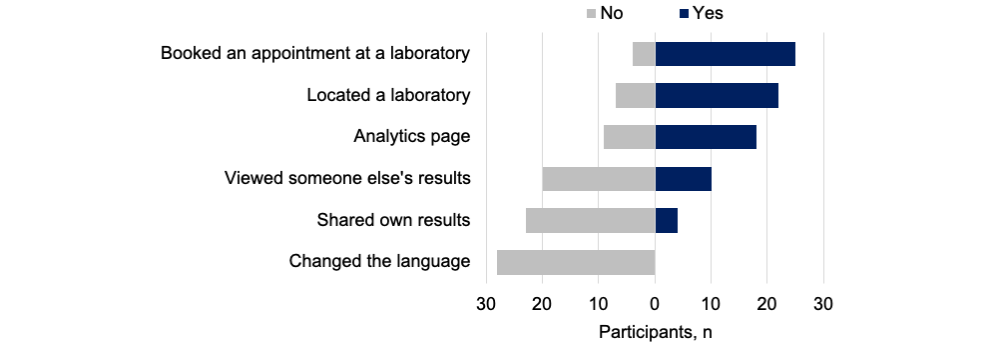
Use of laboratory results portal features (participants indicating “Don’t know” or “Can’t remember” were excluded from this analysis).

We examined which features participants used (Figure 2). Most had booked an appointment on the internet (n=25, 83%) and used the platform to find a laboratory location (n=22, 73%). Fewer participants (n=18, 60%) had used the analytics page.

A patient can choose to share their report with someone else (eg, a family member or caregiver) by clicking a button and inputting an email address. The patient can also customize user viewing privileges to their health data. More participants reported seeing someone else’s laboratory results (n=10, 33%) than sharing their results (n=4, 13%). None reported changing their results to another language.

Respondents were supportive of the addition of a notification feature. Specifically, all but one respondent (n=29, 97%) wanted a notification to let them know when their results were available in the portal.

#### Information in Laboratory Results Portals

**Figure 3:**
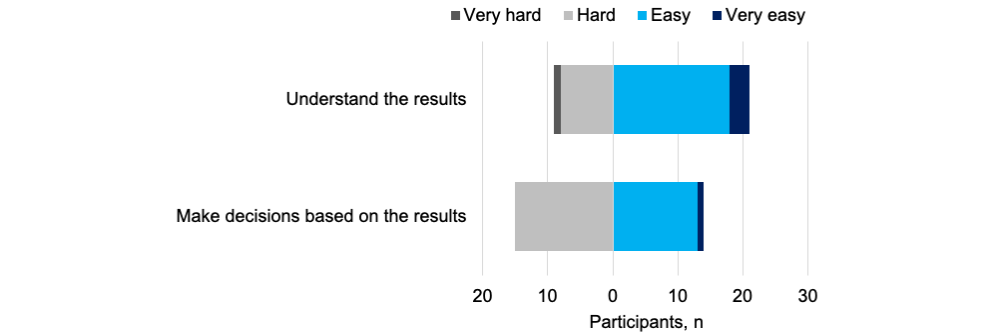
Perceived understanding and ability to use information from laboratory results portals.

Despite rating overall portal information positively (mean 3.5, SD 0.73; Table 1), participants were more critical of specific aspects (Figure 3). In total, 21 (70%) respondents found the information easy or very easy to understand, whereas half (n=15, 50%) found it hard to make decisions based on their results.

#### Laboratory Results Portal Displays

**Figure 4:**
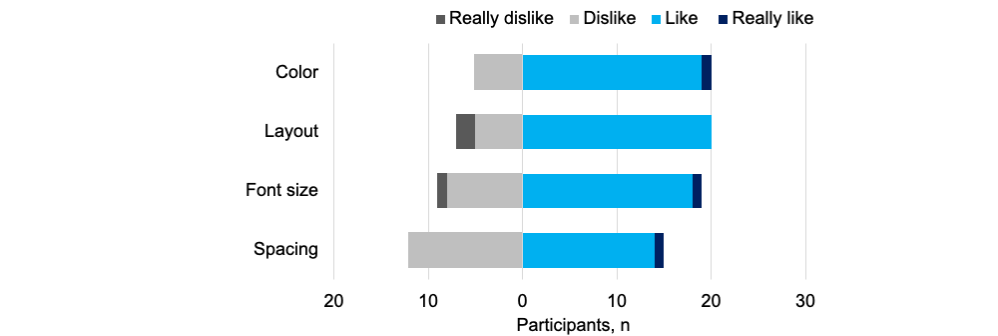
Ratings of visual aspects of laboratory results portal displays.

Display scores were the lowest overall (mean 3.3, SD 0.60; Table 1). We asked participants about 4 display attributes (Figure 4). In total, 25 (83%) respondents liked or really liked the colors of the laboratory results portal displays; 23 (77%) liked or really liked the layout, and 21 (70%) liked or really liked the font size. Spacing seemed most problematic; only 12 (60%) scored spacing positively.

### Qualitative Themes From Open-Ended Responses

#### Overview

We identified four major themes with affinity diagramming: (1) overall access, (2) usability and features, (3) information, and (4) displays. To be included as a theme, we set an a priori reporting threshold of 25% (ie, at least 8, 27%, participants had to articulate the theme for us to include and describe it). If a theme could belong to more than 1 category (eg, participants reported issues with the usability and displays of trend feature), we only report it once for brevity.

#### Overall Access

Many participants (n=11, 37%) liked having access to their laboratory results. Some felt more independent without having to rely upon their health care provider as an “information gatekeeper.” For example, participant 4 wrote, “I love having the option to look it up and not having to wait for my doctor to tell me the results.” Participant 27 wrote, “[It is] helpful to have access to the results, as often doctors don't let you know what the results are.” Other participants believed this access helped them prepare for appointments, engage in clinical discussions, or manage their conditions. For example, participant 23 wrote, “[this is] essential info to doing my part to manage my health challenges.”

#### Usability and Features

We explored participants’ insights about several dimensions of usability, including effectiveness, efficiency, and satisfaction in meeting their goals. Some participants believed laboratory results portals were quick, easy, and straightforward to use, whereas others said the portals were hard to navigate. Many also described difficulty finding specific clinical information.

Some participants mentioned wanting a mobile app rather than using a web browser. For example, participant 30 said, “an app would be amazing.” Several (n=8, 27%) also described usability problems when using laboratory results portals on mobile devices. Some respondents expressed difficulty navigating these portals or understanding the interface user flow on mobile devices. Participant 21 explained, “the site could have a better flow for mobile. Still easy to navigate, but not pleasing to the eye on [the] mobile version.”

Respondents also indicated that the addition of a notification feature to let them know when their results were available in the portals would be beneficial to remind them to check, prevent continuous checking, and save time. For example, participant 10 wrote “after a test, I check repeatedly to see if results are ready. It would be convenient if I just received an email when they [results] are [ready].”

Two-thirds (n=20, 67%) of our respondents commented on the interface’s ability to display laboratory trends. Many liked that portals allowed them to track their values over time or easily recognize when values were improving or deteriorating. However, others (n=13, 43%) described usability issues. For example, participant 3 said, “I find trying to get the trends over time doesn’t seem to be user-friendly.” Many respondents either did not have the capability to view trends, had difficulty viewing them, or were unaware the feature existed.

#### Information Needs

Respondents (n=17, 57%) wanted more descriptions about the tests, reference ranges, information about clinical relevance (eg, creatinine is a measure of kidney function), the meaning of abnormal results, and links to supplementary information. For example, participant 24 wanted “some kind of explanation for people to understand what was being tested and why,” and participant 12 wrote, “[It] would be great to add links to information on what tests are used for and what abnormal results indicate.”

Many participants (n=12, 40%) may have struggled with medical jargon and cited a lack of plain language explanations. Additionally, participants (n=8, 27%) wanted to know what acronyms stood for by providing definitions or including a glossary within the laboratory results portal. For example, participant 17 said, “spell out any abbreviations of test info,” and participant 1 wrote, “I often know what type of tests are ordered, but don't what the items associated with test means – i.e., under haematology, what is MCV? MCH? MCHC?”

#### Displays

Participants complained about attributes of the data displays, including how abnormal values are rendered, use of color, and font size. Many participants (n=17, 57%) wanted out-of-range values to be easier to recognize. For example, respondents suggested emphasizing abnormal values with bold font or highlighting. Participant 10 wrote, “[we need] bold or coloured for abnormal results.” In all, 13 (43%) respondents said color could be improved. They suggested using alternate row shading to make results easier to read and including a color scale or coding scheme for out-of-range test results (eg, red is abnormal and green is within the normal range). When asked about opportunities for improvement, participant 17 wrote, “colour coding: normal, high, low; shade alternating lines to make it easier to read.” In total, 9 (30%) participants wanted larger font or the option to increase the font size.

## Discussion

### Principal Findings

Overall, most respondents in our study valued having access to web-based laboratory results and were likely to recommend laboratory results portals to other consumers [14]. However, their ratings and estimates of portal attributes, including portal usability, informational content, and data displays, suggest there are usability flaws limiting the quality of use and communication effectiveness. Concerning the quality of use, advanced features, such as data trending and analytics, were absent, difficult to find, or rarely used. This raises the question of whether web-based tools are providing deeper insight into chronic illness management or closing knowledge gaps that may occur if patients bypass discussions with their health care team. This seems to be a lost opportunity since graphic user interfaces offer dynamic tools for data visualization, manipulation, and understanding [18-20].

Concerning communication effectiveness, participants indicated they needed additional information to understand their results and contextualize them to their own health status. In the free-text comments, respondents asked for descriptive text, decoded acronyms, the interpretation of results, qualifying information for abnormal values, and hypertext links to additional resources. This is hardly surprising given the importance of clear communication to overcome health literacy barriers, reduce errors, and improve clinical outcomes [21]. Experts have long advocated universal precautions for health communication when interacting with patients in person or on the internet [22,23]. Universal communication precautions are standard methods for discussing technical information to avoid miscommunication and misunderstanding. This is doubly important when communicating with consumers asynchronously. Therefore, portal designers should take the same steps clinicians are expected to take when engaging patients: using plain “everyday” language, including explanations, checking for understanding, and avoiding information overload through progressive disclosure. During synchronous encounters, experts encourage clinicians to use a “teach back” method (ie, asking the patient to explain information in their own words) to confirm understanding [24]. This may pose a novel challenge for web developers. Nevertheless, streaming videos, interactive apps, and artificial intelligence chatbots may offer innovative ways to bridge this gap.

Participant responses to our questions about the data display were more critical. It seemed that color, font, spacing, and layout could all be improved to facilitate more efficient information retrieval and more effective understanding. Again, we anticipated this result given the challenges clinicians face when searching the EHR for key laboratory results [25,26]. In usability studies of contemporary EHR interfaces, clinicians searching for diagnostic information have reported difficulty with navigation, data fragmentation, scale interpretation, search functions, and even readability [26]. Moreover, most laboratory reports intended for consumers look similar to those intended for clinicians. Presenting laboratory results to consumers in formats designed for health care professionals is neither helpful nor safe. It is critical to ensure outputs are clear and safe to use. Otherwise, consumers may overlook important findings [27] or turn to the internet—and other less scrupulous sources—for help interpreting laboratory results [11,28-31].

### Implications of Findings

We believe there is a need for more user experience research with health care consumers to inform the evidence-based design of future patient portals. Developers should test display options to identify what fonts, configurations, colors, and other attributes improve user efficiency, promote action, or reduce errors. This line of inquiry can provide insights into unmet user requirements, new features, and breakthrough innovations. It would also be useful to compare different portals using A/B testing to determine which features or design decisions perform better. For example, do private laboratory portals differ substantially from portals tethered to hospital or clinic EHRs?

We believe that the qualitative responses to our questionnaire offer a base set of requirements for future software development. These quotes can help us to better define problems, understand user needs, and challenge our assumptions. Based on the responses, here is a list of common requirements we believe are important to success: (1) people want timely (ie, quick and on-demand) access to their results; (2) people need access to their data without relying on their providers; (3) people want portals that can be accessed on a browser or personal device; (4) it is important for people to monitor or see trends in their values over time; (5) people want to use results to self-manage their own health conditions; (6) people need assistance interpreting results in the context of their own health; (7) descriptive information should be context sensitive and in plain language; (8) people want clear, easy-to-read displays that highlight abnormal values; (9) abnormal values should include actionable recommendations; and (10) portals should include hypertext links to additional resources or downloads.

These results indicate that in markets where there are alternative options for getting laboratory testing done, it may be prudent for laboratory companies to invest more to attract more customers (consumers). That is, many consumers appreciate laboratory results portals, but the overall user experience could be improved, which could create a competitive advantage. However, if equipping consumers with actionable information leads to them using it to better their health, they may need fewer laboratory tests. Therefore, businesses may actually be deterred from deploying well-designed information this way. However, for countries with public health care, it would be beneficial as it could be used for health promotion, illness prevention, and improved self-management.

### Limitations

There were several limitations to this study. First, we recruited a small sample of experienced portal users. Participants were predominantly women; well educated; English speaking; and living in British Columbia, Canada. Therefore, the perspectives presented here may not be representative of other populations.

Second, participants could have used several different laboratory results portals. We did not attempt to link comments to specific products, designs, or vendors. Therefore, we could not draw any conclusions about specific products or the relative advantages of private-industry laboratory portals or portals tethered to organizational EHRs.

Third, only consumer self-reported data were gathered; our findings are based on subjective perceptions rather than directly observed user performance. Therefore, we may have overestimated the usability and understandability of web-based information—a respondent bias known as the “illusion of fluency” [32]. For example, participants were asked how well they understood the information, but we did not use a specific example and measure their understanding. Related work has shown that even experienced users of laboratory results portals can easily overlook abnormal values [27].

Finally, we only studied the perspectives of users; we did not gather the perspectives of nonusers. Therefore, all participants had experience using one or more laboratory results portals. This represents an important selection bias; users could be very different from nonusers in their personal goals, search strategies, technology literacy, and health literacy. Furthermore, we could not explore all potential deterrents to accessing these systems.

### Conclusions

Through our questionnaire results, we identified the barriers and facilitators to using these systems and highlighted opportunities where such systems could be improved. We identified areas for improvement centered around usability, information, and displays. This study offers health care organizations and health information system developers general recommendations on how to better design these products to align with users’ needs and for optimal use.

## Appendix

Multimedia Appendix 1 Infographic of study findings.

## Abbreviations

EHR: electronic health record
NPS: Net Promoter Score
