# Supplementary material for: Consumers’ Needs for Laboratory Results Portals: Questionnaire Study
Source: JMIR Hum Factors. 2023 Jun 12;10:e42843. doi: 10.2196/42843 (PMC10337333; doi:10.2196/42843)
Supplement: Multimedia Appendix 1 [file humanfactors_v10i1e42843_app1.pdf]

Consumers' Needs for Laboratory (Lab) Results Portals

Findings from a questionnaire with Canadians in British Columbia

By Dr. Helen Monkman & Leah MacDonald

SUMMARY

- Respondents **liked having access** to their lab results.
- **90%** would recommend using them to someone else.
- The **content** and **display** could be improved.
- People want **notifications** when their lab results are ready to view.
- Respondents commented that they want results to be **mobile friendly.**

PARTICIPANTS

30 people responded to the questionnaire

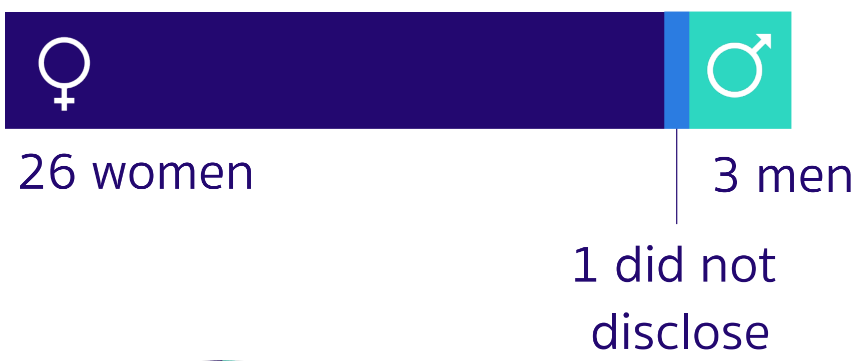

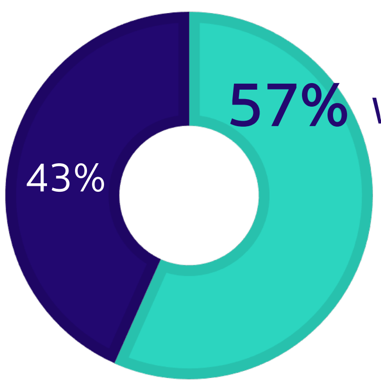

USE OF LAB RESULTS PORTALS

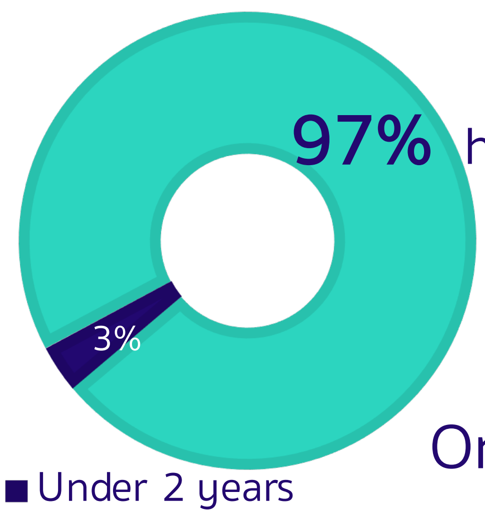

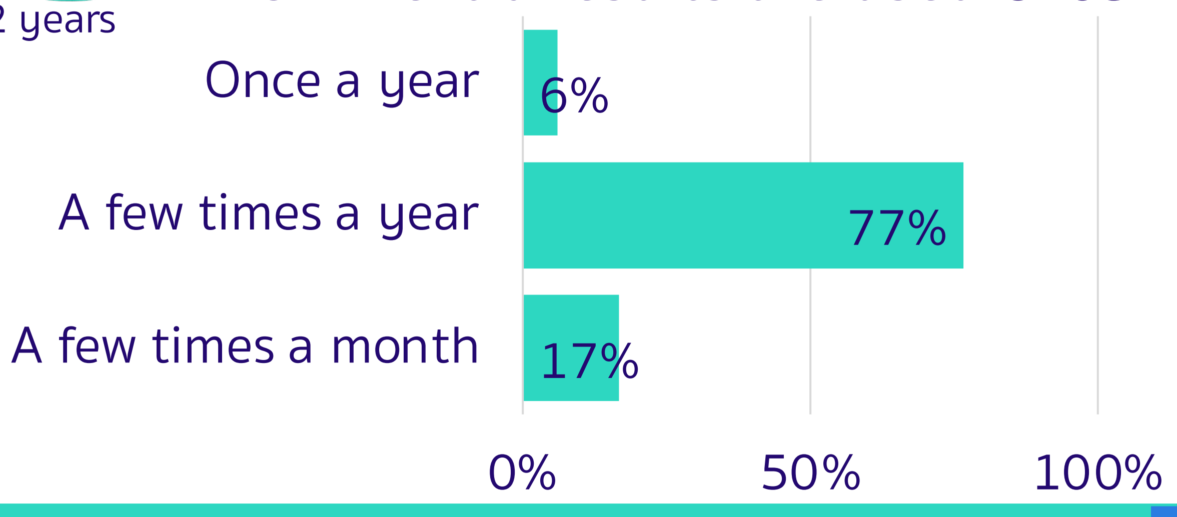

RATINGS

4.5 / 5 stars
Likely to recommend it to someone

3.7 / 5 stars
User friendliness

3.5 / 5 stars
Information in lab results portals

3.3 / 5 stars
Lab results portal **displays**

"I love having the option to look it up and not having to wait for my doctor to tell me the results. I also love being able to compare my results over time, look at all results and have a starting point to research more about the results."

In general, people appreciated having **access to their lab results.**

UNDERSTANDING

The majority (60%) of respondents found it easy to understand their results, but half (50%) found it hard to make decisions based on their results.

Respondents said that **adding links to additional information** would be helpful for understanding medical terms and the significance of **out-of-range** results.

DISPLAY

Most respondents (**66%**) **liked the layout** and (**60%**) **liked the spacing** of their lab results portals.

Respondents said the **font** and **use of color** could be more effective, especially for **out-of-range** results.

NOTIFICATIONS

"After a test, I check repeatedly to see if results are ready. It would be convenient if I just received an email when they are."

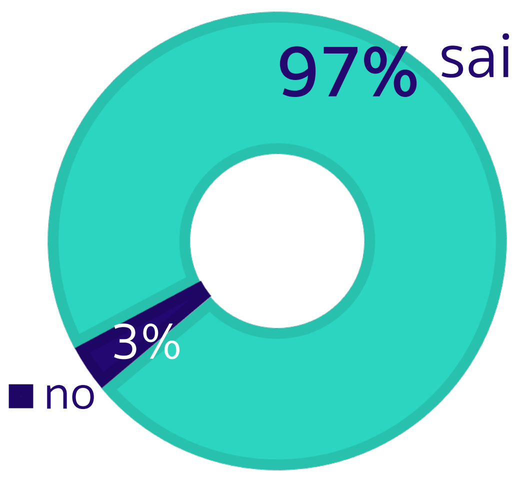

97% said **yes,** they would like **notifications** to:

1. remind
2. prevent continuous checking
3. save time

"Using the website to find test results is easy. The site could have a mode for **mobile** though. Still easy to navigate, but not pleasing to the eye on the mobile version."

MOBILE

Some participants specified that they would like a **mobile option** to view results and get notifications when results are ready

Questions? Please email Dr. Helen Monkman at monkman@uvic.ca
